# Peanut ball during labor to reduce pain and anxiety: A single-blind randomized clinical trial

**DOI:** 10.18332/ejm/220424

**Published:** 2026-05-30

**Authors:** Itzel Osorto Zelaya, Olvin René Reyes Soto, Gabriela Patricia Medina Ventura, Itzel Carolina Fuentes Barahona, Ricardo Arturo Gutierrez-Ramirez

**Affiliations:** 1 Postgraduate Program in Gynecology and Obstetrics, Universidad Nacional Autónoma de Honduras, Tegucigalpa, Honduras; 2 Labor and Delivery Unit, Hospital Escuela, Tegucigalpa, Honduras; 3 Department of Obstetrics and Gynecology, Universidad Nacional Autónoma de Honduras, Tegucigalpa, Honduras

**Keywords:** peanut ball, labor duration, non-pharmacological analgesia, anxiety, labor pain

## Abstract

**INTRODUCTION:**

Pain and anxiety are common during labor. Evidence on non-pharmacological tools like the peanut ball from public hospital settings in Honduras is limited. This study evaluated its efficacy in reducing pain and anxiety.

**METHODS:**

A parallel-group, randomized, single-blind clinical trial was conducted at Hospital Escuela, Honduras, February to July 2025. Nulliparous women in active labor (cervical dilation ≥4 cm) were assigned to an intervention group (standard care + intermittent peanut ball use, n=83) or to a control group (standard care alone, n=80). Primary outcomes were pain and anxiety (Visual Analog Scales). Secondary outcomes included labor duration and oxytocin use. Pre-planned subgroup (no oxytocin) and adjusted analyses were performed.

**RESULTS:**

No significant between-group differences were found in pain (p=0.45), anxiety (p=0.62), or maternal well-being. The active phase duration was shorter in the intervention group, mean=162 minutes (SD=78), compared with the control, mean=252 minutes (SD=138) (mean difference= -90.0 min, 95% CI: -172.37–7.7; p=0.03). This effect persisted in the subgroup without oxytocin (p=0.012) and after adjustment. Oxytocin use was higher in the intervention group (67.5% vs 38.8%; risk ratio, RR=1.74; 95% CI: 1.25–2.42; p=0.001). Cesarean birth and other outcomes were similar.

**CONCLUSIONS:**

The peanut ball shortened active labor but did not reduce maternal pain, anxiety, or oxytocin use within our setting.

**CLINICAL TRIAL REGISTRATION:**

The study is registered on the official website of ClinicalTrials.gov

**IDENTIFIER:**

NCT06811584

## INTRODUCTION

Labor is a unique and transformative experience for women. Therefore, current obstetric care focuses on evidence-based strategies that aim to improve this physiological process in a safe, respectful, and humanized manner. In response to the global focus on quality and respect in maternal care, the International Childbirth Initiative (ICI) has outlined ‘12 Steps to Safe and Respectful Maternity Care’, promoting practices that strengthen women’s autonomy and well-being^1^.

The World Health Organization (WHO), through its 2021 declaration of rights, called to ‘act now for safe and respectful childbirth’, reinforcing the global commitment to ensuring the right to maternal health and a positive, satisfying birth experience. In Honduras, these initiatives translate into efforts to create safe, empathetic, and woman-centered birthing environments for pregnant women^2^.

It has been demonstrated that most healthy women can give birth with minimal interventions, provided that suitable conditions and properly trained professionals are present and understand the physical and emotional needs of laboring women^3^. Among the non-pharmacological strategies to support this process is the use of devices such as the peanut ball, which has been used for decades as a complementary tool to facilitate the labor process^4^.

Pain experienced during labor can be extremely intense, making pain relief an important consideration. Non-pharmacological approaches, including breathing and relaxation techniques, can help reduce pain and discomfort during labor^5^.

Various international studies indicate that the use of peanut balls has shown favorable results, such as pain control, reduced anxiety, improved maternal experience, and optimized obstetric indicators, including reduced cesarean births or instrumental interventions^6,7^.

Beyond potential biomechanical benefits for labor progression, non-pharmacological tools like the peanut ball are theorized to reduce anxiety by promoting a sense of control, facilitating active participation in labor, and providing physical comfort and distraction, which are key components of psychophysiological labor support^8^. This mechanistic rationale underpins our investigation into its effects on both pain and anxiety.

While previous systematic reviews and meta-analyses have evaluated the peanut ball in various settings^9,10^, evidence from public hospitals in low-resource contexts like Honduras remains scarce. Cultural, organizational, and practice differences may influence the effectiveness and implementation of such interventions. Therefore, this study aimed to evaluate the efficacy of the peanut ball in reducing anxiety and pain during labor at the Hospital Escuela, Tegucigalpa, Honduras, to generate context-specific evidence for informing local practice.

## METHODS

### Trial design and study setting

A parallel-group, randomized, single-blind (outcome assessor and data analyst) clinical trial was conducted following the Consolidated Standards of Reporting Trials (CONSORT) statement and its extension for non-pharmacological trials (CONSORT-NPT). The study was carried out at the Hospital Escuela, Tegucigalpa, Honduras, from 1 February to 31 July 2025.

### Participants

Inclusion criteria were: pregnant women aged 18–45 years; gestational age ≥36 weeks; singleton pregnancy with cephalic presentation; nulliparous; cervical dilation in active labor (cervical dilation ≥4 cm and regular, painful contractions); and written informed consent. Exclusion criteria were: pre-pregnancy body mass index (BMI) >30 kg/m²; multiple gestation; high-risk pregnancy (e.g. preeclampsia, fetal growth restriction, placenta previa); contraindications to vaginal delivery; known major fetal anomalies; musculoskeletal conditions preventing ball use; diagnosed mental illness; and multiparity.

### Interventions

*Experimental group*

Participants received standard obstetric care plus the peanut ball intervention. The peanut ball is an oblong, peanut-shaped physiotherapy ball designed to be placed between a laboring person’s knees while in a lateral or semi-sitting position, with the aim of promoting pelvic alignment and fetal descent. Starting at admission in active labor, the ball was used in 30-minute sessions every 2 hours, with trained staff assisting participants in adopting recommended positions (lateral decubitus, semi-sitting, or Taylor position). Use was discontinued at full cervical dilation (10 cm) or at the onset of the second stage (active pushing).

*Control group*

Participants received standard obstetric care alone, which included continuous fetal heart rate monitoring, ambulation ad libitum, intravenous analgesia upon request, and oxytocin augmentation per institutional protocols for labor dystocia (arrest of dilation). The decision to administer oxytocin was made independently by the managing clinician based solely on clinical indications and was not part of the study intervention.

*Provider training*

All participating healthcare staff completed a 4-hour standardized training on peanut ball use, positioning, and contraindications.

### Outcomes

*Primary outcomes*

Primary outcomes were pain intensity (Visual Analog Scale, VAS 0–10, a 10-cm scale where 0 represents ‘no pain’ and 10 represents ‘the worst pain imaginable’) and anxiety (Modified VAS for Anxiety, VAS-A 0–10), measured at three time points: at admission/baseline (cervical dilation 4–5 cm), during active labor (5–7 cm), and during transition (8–10 cm). Pain-related anxiety was also assessed postpartum using the PASS-20 scale (Spanish version).

*Secondary outcomes*

Secondary outcomes were: active phase labor duration (minutes); obstetric interventions (oxytocin use, mode of delivery, episiotomy, perineal tear, postpartum hemorrhage); maternal well-being (BMSP-2 scale); and fear of childbirth (W-DEQ Parts A and B).

*Neonatal outcomes*

Were defined as a composite of: 1-minute and 5-minute Apgar scores <7, admission to the neonatal intensive care unit (NICU), or requirement for respiratory support.

*Operational definitions*

Postpartum hemorrhage was defined as estimated blood loss >500 mL after vaginal delivery; and perineal tears were classified using standard obstetric criteria.

### Measured variables and potential confounders

Demographic and baseline variables collected included maternal age (years, analyzed continuously and categorically), geographical origin (rural/urban), education level (categorized into 8 levels: none, incomplete primary, completed primary, incomplete secondary, complete secondary, incomplete university, college graduate), and marital status (single, married/common-law union). Obstetric history variables comprised gestational age at delivery (weeks), number of previous pregnancies, deliveries, cesarean sections, abortions, and ectopic pregnancies. Intrapartum variables included cervical dilation at enrollment, status of chorioamniotic membranes (intact/ruptured), and use of oxytocin augmentation.

Oxytocin augmentation was identified *a priori* as a key potential confounder for the analysis of labor duration, given its significant between-group difference following randomization and its well-established physiological effect on uterine contractility and labor progression^4^. Education level, which showed a baseline imbalance between groups, was also considered a potential confounder for secondary social and psychological outcomes.

### Sample size

The sample size was calculated for the primary outcome of pain intensity, measured on a 0–10 Visual Analog Scale (VAS). The parameters for this calculation were based on a systematic review and meta-analysis of non-pharmacological interventions for labor pain. A review reported a mean reduction of approximately 1.7 points on the VAS for childbirth ball exercises, with a pooled standard deviation of approximately 2.5 points among control groups. Therefore, assuming a mean difference of 1.5 points, a common standard deviation of 2.5, a two-sided alpha of 0.05, and a power of 80%, a minimum number of 79 participants per group was required. Using G*Power software (version 3.1), the calculation for an independent t-test confirmed this sample size. A total of 163 women were recruited to account for a potential attrition rate of up to 3%.

We acknowledge that this sample size provides adequate power for the continuous primary outcome but is underpowered to detect clinically meaningful differences in less frequent binary secondary outcomes, such as mode of delivery or postpartum hemorrhage.

### Randomization and allocation concealment

An independent statistician generated the 1:1 allocation sequence using variable block sizes. Sequentially numbered, opaque, sealed envelopes were prepared. The head of the labor unit – who was not involved in participant recruitment or outcome assessment – stored the envelopes and performed group assignment after obtaining informed consent. Allocation concealment was maintained until the moment of intervention.

### Blinding

Due to the nature of the intervention, blinding of participants and care providers was not possible. However, outcome assessors and data analysts remained blinded to group assignment throughout the study.

### Implementation and adherence

Trained staff recorded each intervention session in a daily log. Weekly audits by the research team ensured protocol compliance. Adherence was defined as completion of ≥80% of scheduled peanut ball sessions.

### Statistical analysis

Data were analyzed using SPSS v.26.0. Continuous variables were compared using Student’s t-test or the Mann-Whitney U test, as appropriate; categorical variables were compared using chi-squared or Fisher’s exact test. Repeated-measures ANOVA with Greenhouse-Geisser correction assessed pain and anxiety trends over time. Given the baseline imbalance in education level (Table 1) and the significant between-group difference in oxytocin administration (a key potential confounder for labor duration), a *post hoc* adjusted analysis was conducted. The duration of the active phase was modeled as the dependent variable in a multivariable linear regression, with group assignment as the independent variable, adjusting for education level and oxytocin administration as covariates. A pre-planned subgroup analysis was specified to evaluate the effect of the peanut ball in the cohort of women who progressed spontaneously, i.e. those who did not receive oxytocin augmentation for labor dystocia. Data are presented as mean ± standard deviation (SD). For binary outcomes, risk ratios (RR) with 95% confidence intervals (CIs) were calculated using log-binomial regression (or Poisson regression with robust variance where log-binomial did not converge), as they provide a more intuitive measure of association for this study design than odds ratios. All analyses followed the intention-to-treat (ITT) principle. A p<0.05 was considered statistically significant.

**Table 1:** Baseline sociodemographic and obstetric characteristics of participants in a single-blind randomized clinical trial of peanut ball use during labor, Hospital Escuela, Honduras, 2025 (N=163)

*Characteristics*	*Intervention* *(N=83)* *n (%)*	*Control* *(N=80)* *n (%)*	*p*
**Age** (years), mean (SD) [95% CI]	22.8 (5.2) [21.7–24.0]	24.8 (6.0) [23.4–26.1]	0.07^a^
**Age** (years)			0.18^b^
<18	17 (8.4)	3 (3.8)	
18–35	75 (90.4)	73 (91.3)	
>35	1 (1.2)	4 (5.0)	
**Origin**			0.59^b^
Rural	26 (31.3)	25 (31.3)	
Urban	57 (68.7)	54 (67.5)	
**Education level**			0.01^b^
None	4 (4.8)	1 (1.3)	
Incomplete primary school	16 (19.3)	8 (10.0)	
Completed primary school	9 (10.8)	4 (5.0)	
Incomplete secondary school	20 (24.1)	22 (27.5)	
Complete secondary education	19 (22.9)	38 (47.5)	
Incomplete university studies	6 (7.2)	1 (1.3)	
College graduate	9 (10.8)	5 (6.3)	
**Marital status**			0.56^b^
Single	14 (16.9)	12 (15.0)	
Married/common-law union	69 (83.1)	67 (83.8)	
**GA at delivery,** mean (SD) [95% CI]	39.0 (1.2) [38.7–39.2]	38.9 (1.2) [38.6–39.2]	0.86^a^
**Previous pregnancies**			0.96^a^
0	4 (4.8)	6 (7.5)	
1	59 (71.1)	54 (67.5)	
2	17 (20.5)	16 (20.0)	
≥3	2 (2.4)	4 (5.0)	
**Previous deliveries**			0.33^a^
0	73 (88.0)	72 (90.0)	
1	5 (6.0)	3 (3.8)	
≥2	1 (1.2)	0 (0.0)	
**Previous cesarean births**			0.32^a^
0	78 (94.0)	83 (100.0)	
1	1 (1.2)	0 (0.0)	
**Previous abortions**			0.46^a^
0	61 (73.5)	62 (77.5)	
1	17 (20.5)	11 (13.8)	
≥2	2 (2.4)	3 (3.8)	
**Previous ectopic pregnancies**			0.6^a^
0	77 (92.8)	73 (92.8)	
1	2 (2.4)	3 (3.8)	

GA: gestational age (weeks).

a Mann-Whitney U test.

b Chi-squared test.

## RESULTS

**Figure 1:**
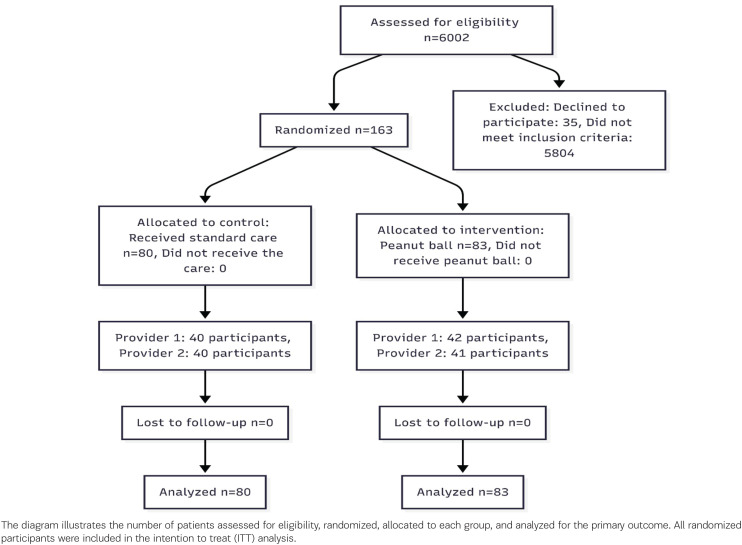
CONSORT flowchart of participant enrollment, allocation, and analysis in a randomized clinical trial evaluating the peanut ball during labor, Hospital Escuela, Honduras, February–July 2025

A total of 163 pregnant women were recruited and randomly assigned: 83 to the intervention group (standard care plus peanut ball) and 80 to the control group (standard care alone) (Figure 1). Protocol adherence was high, with 95% of participants in the intervention group completing ≥80% of scheduled peanut ball sessions. Recruitment exceeded the minimum calculated sample size of 79 per group to account for potential attrition, which did not occur.

### Baseline characteristics

Baseline sociodemographic and obstetric characteristics are presented in Table 1. The mean age was not statistically different between the two groups (22.8 ± 5.2 vs 24.8 ± 6.0 years, p=0.07). A significant between-group difference was found in education level (χ²=18.48, p=0.01), with a higher percentage of participants in the control group having completed secondary education (47.5% vs 22.9%). No significant differences were observed in place of origin, marital status, or baseline obstetric history (all p>0.05).

### Primary outcomes

*Labor duration and oxytocin use*

As shown in Table 2, the duration of the active phase of labor was significantly shorter in the intervention group (162 ± 78 minutes) compared to the control group (252 ± 138 minutes; mean difference= -90.0 min, 95% CI: -172.3 – -7.7; p=0.03). Oxytocin augmentation was administered significantly more frequently in the intervention group (67.5% vs 38.8%; p=0.001).

**Table 2:** Intrapartum characteristics and obstetric outcomes of participants in a single-blind randomized clinical trial of peanut ball use during labor, Hospital Escuela, Honduras, 2025 (N=163)

*Characteristics*	*Intervention* *(N=83)* *n (%)*	*Control* *(N=80)* *n (%)*	*p*
**Cervical dilation** (cm), median (range)	4 (0–8)	5 (0–8)	0.60^a^
**Chorioamniotic membranes**			0.18^b^
Intact	47 (56.6)	37 (46.3)	
Ruptured	36 (43.4)	43 (53.8)	
**Duration of the active stage** (minutes), mean (SD) [95% CI]	162 (78) [120–204]	252 (138) [204–300]	**0.03^a^**
**Type of delivery**			0.42^b^
Vaginal birth	74 (89.2)	68 (85.0)	
Cesarean birth	9 (10.8)	12 (15.0)	
Use of oxytocin	56 (67.5)	31 (38.8)	0.001^b^
Episiotomy	35 (42.2)	34 (42.5)	0.97^b^
Perineal tears	42 (50.6)	31 (38.8)	0.12^b^
Postpartum hemorrhage	2 (2.4)	3 (3.8)	0.63^b^

a Mann-Whitney U test.

b Chi-squared test.

*Pain and anxiety during labor*

No significant differences were found between groups in pain or anxiety levels at any measurement point (Table 3). Repeated-measures ANOVA showed no significant group-by-time interaction for pain (p=0.633) or anxiety (p=0.554). Scores on the PASS-20, BMSP-2, and W-DEQ scales also showed no significant differences (all p>0.05).

**Table 3:** Pain and anxiety outcomes according to study group in a single-blind randomized clinical trial of peanut ball use during labor, Hospital Escuela, Honduras, 2025 (N=163)

*Characteristics*	*Intervention (N=83)*	*Control (N=80)*	*p*
**Pain (VAS),** mean (SD) [95% CI]*			
Assessment 1	7.2 (1.9) [6.8–7.6]	7.1 (2.2) [6.6–7.7]	0.85^a^
Assessment 2	7.7 (1.4) [7.3–8.4]	7.6 (1.7) [7.2–8.9]	0.92^a^
Assessment 3	8.4 (1.4) [7.8–8.4]	7.3 (1.6) [6.9–7.6]	0.25^a^
**Anxiety (VAS-A),** mean (SD) [95% CI]*			
Assessment 1	7.1 (1.7) [6.7–7.4]	6.9 (2.1) [6.4–7.5]	0.65^a^
Assessment 2	7.0 (1.4) [6.7–7.4]	7.6 (1.6) [6.6–7.5]	0.99^a^
Assessment 3	7.3 (1.6) [7.0–7.6]	7.6 (1.2) [7.3–7.8]	0.21^a^
**PASS20,** mean (SD) [95% CI]	70.1 (13.6) [67.1–73.0]	69.1 (19.8) [64.7–73.6]	0.74^b^
**BMSP2 scale,** median (range)	169.0 (90–191)	165 (40–203)	0.52^c^
**W-DEQ part A,** median (range)	100.0 (72–232)	102.0 (39–160)	0.96^c^
**W-DEQ part B,** median (range)	98 (68–151)	97 (30–140)	0.42^c^

* Pain assessments (VAS) and anxiety assessments (VAS-A) were conducted at the following times: Assessment 1: 30 minutes after the start of peanut ball use (or the start of monitoring in the control group) and with cervical dilation <5 cm; Assessment 2: With cervical dilation between 5–7 cm; Assessment 3: With cervical dilation between 8–10 cm. PASS20: Perinatal Anxiety Screening Scale. BMSP2 Scale: Brief Psychological Distress Measurement Scale, version 2. W-DEQ: Wijma Delivery Expectancy/Experience Questionnaire.

a Mann-Whitney U test.

b Student’s t-test.

c Chi-squared test.

The results of the multivariable linear regression analysis, adjusting for oxytocin use and education level, are presented in Table 4. The use of the peanut ball remained significantly associated with a shorter active phase duration (β= -85.4 minutes, 95% CI: -167.8 – -3.0; p=0.042).

**Table 4:** Multivariable linear regression analysis for the duration of the active phase of labor (minutes) in a randomized trial of peanut ball use, Hospital Escuela, Honduras, 2025 (N=163)

*Variables*	*Coefficient (β)*	*95% CI*	*p*
Intervention group (vs control)	-85.4	(-167.8 – -3.0)	0.042
Oxytocin use (vs non-use)	92.1	(25.5–158.7)	0.007
Education level high (vs low)	-15.2	(-78.3–47.9)	0.635

The dependent variable is the duration of the active phase in minutes. The model presents results from a multivariable linear regression. The coefficient (β) represents the adjusted mean difference in minutes. Educational level was dichotomized (High: complete secondary education or higher; Low: lower than complete secondary education) for this analysis.

### Subgroup analysis

*Spontaneous labor progression (no oxytocin augmentation)*

In the pre-planned subgroup analysis of women with spontaneous labor progression (i.e. those who did not receive oxytocin, n=76), the duration of the active phase remained significantly shorter in the intervention group (n=27; 162 ± 83.4 minutes) compared to the control group (n=49; 256.8 ± 139.8 minutes) (p=0.012).

Within this same subgroup, no statistically significant differences were observed between the intervention and control groups in any measure of pain or anxiety: mean VAS pain scores (7.2 vs 8.0; p=0.347), mean VAS-A anxiety scores (6.9 vs 7.2; p=0.817), PASS-20 (69.9 vs 69.3; p=0.897), BMSP-2 (161.3 vs 166.6; p=0.463), W-DEQ part A (107.2 vs 110.3; p=0.651), or W-DEQ part B (94.3 vs 95.2; p=0.724).

## DISCUSSION

The principal findings indicate that the intervention did not reduce maternal pain or anxiety levels, nor did it improve maternal well-being or reduce fear of childbirth, as measured by validated scales at multiple time points. However, the use of the peanut ball was associated with a statistically significant reduction in the duration of the active phase of labor. A notable and unexpected finding was the significantly higher rate (RR=1.74, p=0.001) of oxytocin augmentation in the intervention group. These results present a complex picture that aligns with some aspects of the existing evidence while contrasting with others^9-13^.

The results of this study are partially consistent but also contrast with prior evidence. Our finding of no significant reduction in pain contrasts with a recent meta-analysis by Makvandi et al.^14^, which found that peanut ball use significantly reduced labor pain. Similarly, other studies have reported lower pain scores in intervention groups at advanced cervical dilation^8,12-16^. Furthermore, a meta-analysis ranking non-pharmacological interventions placed birth ball exercises as moderately effective for pain relief^13^.

The discrepancy between our results and those of prior studies may be attributable to differences in study protocols, population characteristics (such as cultural perceptions of pain), timing and frequency of ball use, or the specific outcome measures employed.

In contrast, our finding of a shorter active phase aligns with the existing literature. The mean reduction of approximately 90 minutes in our study is consistent with meta-analytic evidence, such as the review by Delgado et al.^17^, which reported an average reduction of 53–87 minutes in the first stage of labor among women using a peanut ball, particularly with epidural analgesia. The persistence of this effect in our pre-planned subgroup analysis of women with spontaneous labor progression (i.e. without oxytocin) and in a *post hoc* analysis adjusting for confounders, strengthens the likelihood that it is related to the intervention itself, potentially through biomechanical facilitation of fetal descent and pelvic alignment.

Regarding the mode of delivery, the observed cesarean delivery rate was 10.8% in the intervention group compared to 15.0% in the control group (RR=0.72). Although this difference was not statistically significant in our underpowered sample, the direction and magnitude of the effect are consistent with the pooled risk ratio (RR=0.82) reported in a meta-analysis of six trials^18^. This suggests a potential protective effect against cesarean delivery that warrants investigation in larger, adequately powered trials. The significantly higher use of oxytocin in the intervention group (67% vs 38%) is a complex and unexpected finding that challenges a straightforward interpretation. It is possible that clinicians, observing a slower-than-expected progress in the control group, adhered more strictly to protocols for augmentation, whereas in the intervention group, the use of the ball may have been perceived as an alternative or adjunct, yet still triggered augmentation at a similar or higher threshold. This finding underscores the intricate interplay between non-pharmacological tools and clinical decision-making in real-world settings.

The subgroup analysis of women with spontaneously progressing labor reinforced the main findings of the study. By excluding participants with oxytocin-induced labor, the absence of significant differences in pain, anxiety, fear of childbirth, and maternal well-being persisted, suggesting that the use of the peanut ball does not significantly alter the subjective experience of labor, even in the context of physiological progression. This finding is relevant as it indicates that the potential benefit of the intervention in shortening labor duration is not necessarily mediated by an improvement in pain perception or the emotional state of the mother^19-21^.

### Strengths and limitations

Its design as a randomized controlled trial represents the gold standard for evaluating the efficacy of an intervention. We maintained rigorous allocation concealment and adhered to a pre-defined protocol, which enhances internal validity. The study achieved its target sample size for the primary outcome without any loss to follow-up, preserving statistical power for continuous endpoints. Furthermore, we employed validated and widely used instruments (VAS, VAS-A, PASS-20, W-DEQ, BMSP-2) to measure subjective outcomes, improving the reliability and reproducibility of our assessments. The intervention itself (the peanut ball) proved to be feasible and safe in this setting, with no reported adverse events related to its use, supporting its potential for integration into routine care as a low-cost, non-pharmacological tool^22,23^.

Several limitations must be considered when interpreting our findings. First, despite randomization, baseline imbalance in education level was observed, and more critically, a significant disparity in oxytocin augmentation arose between groups. Although we addressed this through pre-planned subgroup and *post hoc* adjusted analyses, the possibility of residual confounding from unmeasured factors (e.g. subtle cervical dynamics or unassessed psychosocial factors) cannot be entirely ruled out. Second, the nature of the intervention made blinding of participants and healthcare providers impossible, which may have introduced performance bias. Furthermore, the reliance on self-reported measures for pain and anxiety could introduce information bias or misclassification. Third, while the sample size was adequate for the primary continuous outcome of pain, it was underpowered to detect clinically meaningful differences in important binary obstetric outcomes, such as mode of delivery or postpartum hemorrhage. Finally, the conduct of the study in a single tertiary-care center may limit the generalizability of our findings to other settings.

## CONCLUSIONS

This randomized trial conducted in a public hospital found that using a peanut ball during labor did not reduce maternal pain, anxiety, or fear of childbirth. However, its use was associated with a clinically meaningful shortening of the active phase of labor, an effect that persisted after accounting for oxytocin use. The intervention was safe and well-tolerated. The concurrent finding of increased oxytocin augmentation in the intervention group is unexpected and requires further study to understand its determinants and implications. While the peanut ball may be a useful, low-cost tool to facilitate labor progress in similar settings, our results do not support its use for improving the subjective emotional experience of birth within our setting. Future research should prioritize larger, multicenter trials to confirm its effect on obstetric outcomes and to better understand its integration into clinical workflows alongside pharmacological interventions.

## Data Availability

The datasets used and/or analyzed during the current study are available from Mendeley Data [Gutierrez-Ramirez, Ricardo Arturo (2025), ‘BAM Trial’, Mendeley Data, V1, doi:10.17632/wdtxjnfm5g.1]
